# GenomeViz: visualizing microbial genomes

**DOI:** 10.1186/1471-2105-5-198

**Published:** 2004-12-15

**Authors:** Rohit Ghai, Torsten Hain, Trinad Chakraborty

**Affiliations:** 1 Institute of Medical Microbiology, Faculty of Medicine, Justus-Liebig-University, Frankfurter Strasse 107, D-35392 Giessen, Germany

## Abstract

**Background:**

An increasing number of microbial genomes are being sequenced and deposited in public databases. In addition, several closely related strains are also being sequenced in order to understand the genetic basis of diversity and mechanisms that lead to the acquisition of new genetic traits. These exercises have necessitated the requirement for visualizing microbial genomes and performing genome comparisons on a finer scale. We have developed GenomeViz to enable rapid visualization and subsequent comparisons of several microbial genomes in an interactive environment.

**Results:**

Here we describe a program that allows visualization of both qualitative and quantitative information from complete and partially sequenced microbial genomes. Using GenomeViz, data deriving from studies on genomic islands, gene/protein classifications, GC content, GC skew, whole genome alignments, microarrays and proteomics may be plotted. Several genomes can be visualized interactively at the same time from a comparative genomic perspective and publication quality circular genome plots can be created.

**Conclusions:**

GenomeViz should allow researchers to perform visualization and comparative analysis of up to eight different microbial genomes simultaneously.

## Background

Current efforts in genome sequencing have led to a rapid increase in the number of microbial genome sequences. A total of 522 ongoing microbial genome projects are listed in the GOLD database [1] and while 167 microbes have been completely sequenced. These sequencing projects now include several bacterial pathogens and different isolates of the same bacterial species that differ with respect to virulence and physiology. Thus, complete and partial genome sequences of a number of closely related species/strains from various genera such as *Escherichia*, *Bacillus*, *Helicobacter*, *Mycobacterium*, *Streptococcus*, *Staphylococcus *and *Listeria *are available. Genomic data too, is diverse, ranging from COG functional classification data [2], genomic islands [3,4], expression data from microarrays and proteomics, GC skew, AT skew, GC%, to whole genome alignments. Such rapid increase in genomic information necessitates the development of tools that offer rapid and convenient visualization capabilities. Furthermore, it is important to contrast and compare data deriving from several different sources (computational, genomic, proteomic) to have a better understanding of genome function.

Several genome visualization tools have been developed in the last few years. The **Microbial Genomes Viewer **[5] offers a good online solution to genomic visualization, allowing flexibility in using one's own data. However, the plot is not very interactive and provides no undo facility as once a mistake is made one has to recreate the entire plot. **GenoMap **[6] can be used to create circular genome plots. Although the visualization is helpful, only limited interaction is possible with the resulting plot. **GenomeAtlas **[7] provides picture-based structural DNA analysis for a large number of genomes via a web-interface. **GenomePlot **[8] also provides a method to render chromosome wheel plots using tab-delimited input files, although it lacks the interactivity with the pictures and requires a rather specific input file format that may have to be customized for each genome. **BugView **[9] is another application that allows comparative analysis of microbial genomes, however it allows only two genomes to be viewed and compared simultaneously. The linear plots are useful and offer much flexibility but the circular plots are static. **Genome2D **[10] offers useful visualization options for data visualization and integration of several algorithms for a single genome at a time. Artemis [11] and ACT [12] are convenient programs for visualizing single genomes or comparing multiple genomes on linear scales.

## Implementation

GenomeViz has been programmed in ActiveTcl. ActiveTcl [13] is available freely from ActiveState. PERL [14] is needed to run the scripts available with GenomeViz. This is usually installed on Linux and Solaris systems but can also be freely downloaded. GenomeViz works only on Unix-based platforms and has been tested on Linux and Solaris operating systems. Currently, it does not work on Windows because of a bug in the Tcl library on Windows which causes narrow arcs on a canvas to be drawn incorrectly. We recommend a minimum of 512 MB RAM to run the program.

## Results and discussion

**Figure 1:**
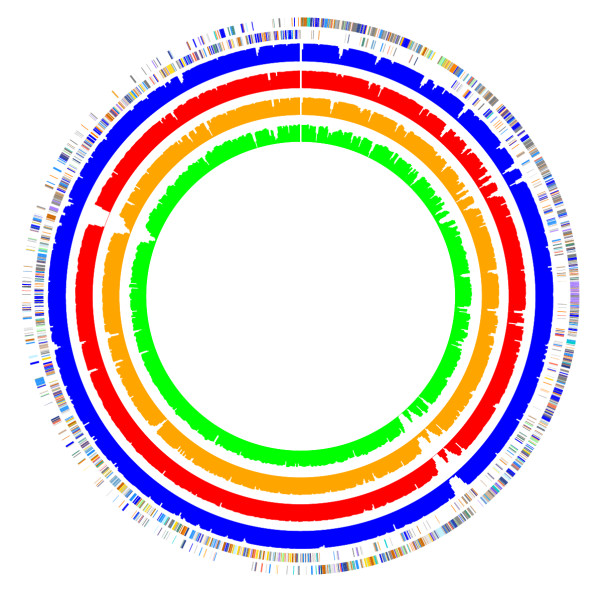
**Whole genome alignments of five *Listeria *strains/species. **From outside to inside: *L. monocytogenes *EGDe serovar 1/2a COG categories (outer two circles), *L. monocytogenes *F6854 serovar 1/2a (blue, 133 contigs), *L. monocytogenes *F2365 serovar 4b (red, whole genome), *L. monocytogenes *H7858 serovar 4b (orange, 180 contigs) and *L. innocua *(innermost, green, whole genome). All genomes were aligned separately to *L. monocytogenes *EGDe with AVID. Sequence data for strains *L. monocytogenes *strains F6854 and H7858 was obtained from The Institute for Genomic Research [17].

GenomeViz uses the concept of "tags" which may be applied to groups of genes for classification-type data. A tag file is tab-delimited text file of three columns. It has the "tags", their colors, and their brief descriptions. A pre-prepared tag file for the COG functional categories is available for immediate use. The map file has all the information required to create the plot (gene name, strand, start and end in genome, annotation, and the tag for the gene). It is also a tab-delimited text file. Both file formats (tag and map) are easy to manipulate in a spreadsheet application like Microsoft Excel. However, care must be taken while manipulating data in such applications since errors may creep in the data as demonstrated by Zeeberg et al. [15]. The map file alone is sufficient for plotting numerical data, but both the map and tag files are needed to plot classification-type data. Data type (qualitative or quantitative) is automatically detected from the map file. A PERL script "tagit" is also available for "tagging" a particular set of genes with user-defined tags. Another script, "avid2viz" is also available which reformats whole genome alignments created by the AVID program [16] to a map file format that can be visualized in GenomeViz (Figure 1).

In order to minimize initial difficulty that users may encounter in creating their own map files, we provide pre-prepared map files for over a hundred genomes. Of these nearly seventy genomes are loaded with the COG classification scheme and may be used immediately. The program also performs checks on the input map file for possible formatting errors and attempts to indicate location of errors (if any) before creating the plot. Several types of plots may be created; on either single or double strands and color gradients and line-graphs are available for numerical data. Once the plots are done, mouse-over on any gene immediately displays associated information from the map file in a display area.

Using GenomeViz, it is also possible to search, highlight and retrieve genes of interest. Each loaded genome may be queried separately. Regular expression searches are fully supported and results are highlighted in the genome. For instance, the simple expression ribosomal|ribosome will mark in color all ribosomal proteins in any genome and retrieve all the information for these genes from the map file. The "|" operator is the standard OR operator in Tcl expressions. Genes involved in iron metabolism/regulation which are usually annotated with keywords like ferrous, ferric or iron may be retrieved with the expression "ferric|ferrous|iron". The results can be saved as a text file. Users can also use their own annotations to visualize and query their genome of interest provided these annotations are available in the map file format. It is also possible to display, in different colors, the results of different queries on the same genome by changing the search color before performing a search. This will enable visualization of, for instance, the distribution of genes/operons involved in iron and zinc metabolism/regulation separately.

**Figure 2:**
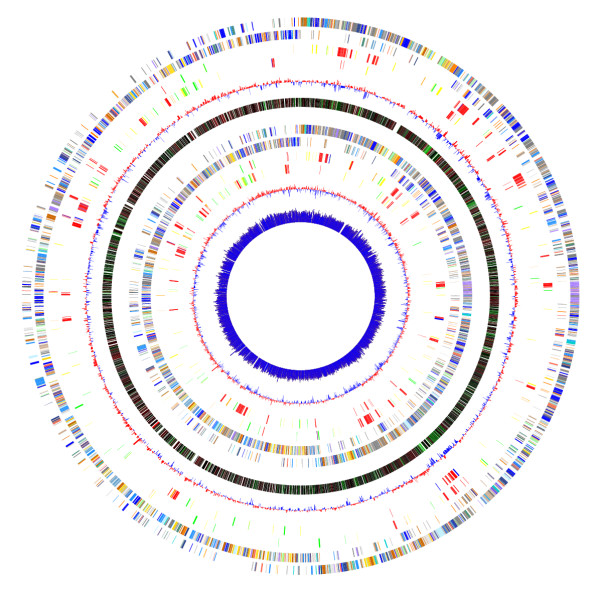
**A typical image generated by GenomeViz. **From outside to inside: *Listeria monocytogenes *COG categories (two circles), horizontally transferred gene categories in *L. monocytogenes *identified using SIGI (two circles), mean centered GC% of *L. monocytogenes *genes (red-above mean, blue-below mean, one circle), GC% gradient (red-high GC%, green-low GC%, one circle), *Listeria innocua *COG categories (two circles), horizontally transferred gene categories in *L. innocua *identified using SIGI (two circles), mean centered GC% of L. innocua genes (red-above mean, blue-below mean, one circle), GC% of *L. innocua *genes shown as a line graph (innermost circle). When created in GenomeViz, this image is fully interactive and any plotted circle may be queried. It also shows the different ways in which qualitative or numerical data may be plotted. Differences in the horizontally transferred genes in the two *Listeria *species may be examined and related to GC content in the region.

**Figure 3:**
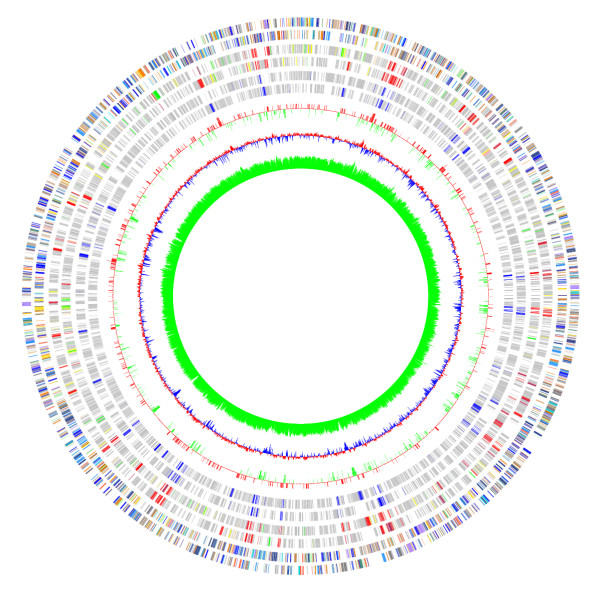
**Comparing data from different sources using GenomeViz.**The figure shows a comparison of the distribution of horizontally transferred genes in *Escherichia coli *K12 compiled from three different sources. From outside to inside: *Escherichia coli *K12 COG categories (two circles), genes identified by SIGI (two circles), genes listed in the Horizontal Gene Transfer Database [18] (two circles), standard deviations of genes identified by IslandPath (single circle, red +ve, green -ve), mean centered GC content of the genome (red: above mean, blue: below mean), GC content of the genome again as a single-sided line plot (green).

A 'Select COGs' option enables one to retrieve all genes from a particular COG category, e.g. "Cell division and Chromosome partitioning" or "Transcription". Each loaded genome can thus be queried separately. Usually, this is more useful when using a special tag file (CogsGrayScale.tag) that colors all genes as "grey", so that a neutral background is available for highlighting the distribution of genes of a particular COG category over the entire genome. Categories of interest can be highlighted in different colors simply by changing the selection color before selecting the category. Results of each query are also displayed in a text box from where they may be saved as a text file. Thus, GenomeViz allows a rapid overview of the similarities in distribution of various functional categories in closely related genomes (Figure 2). It is also possible to visualize differences/similarities in data derived from various different sources e.g. horizontally transferred genes (Figure 3).

Several options are available for printing the circular plot. The graphics can be directly sent to the printer or saved to a PostScript file and read by standard graphics programs. A number of page size options are available and extra large plots spanning many pages may also be printed. A detailed program manual is available with notes on installation, usage and examples.

## Conclusions

We describe a rapid and convenient application GenomeViz for simultaneous visualization and comparison of varied genomic data from several microbial genomes. Future updates for software and data will be available from the project home page.

## Availability and requirements

• **Project name: **GenomeViz

• **Project home page: **

• **Operating system(s): **Linux, Solaris, Unix

• **Programming language: **Tcl/Tk

• **Other requirements: **ActiveTcl, PERL

• **License: **Free for academic use

• **Any restrictions on use by non-academics: **Contact corresponding author for a license.

## List of abbreviations used

COG: Clusters of Orthologous Groups

SIGI: Score-based Identification of Genomic Islands

## Authors' contributions

RG conceived the program, wrote and tested it, prepared the manuals and the website. TC oversaw the entire development process. TH offered suggestions on program features. RG and TC prepared the manuscript. All authors read and approved of the final manuscript.

## References

[B1] Bernal A, Ear U, Kyrpides N (2001). Genomes OnLine Database (GOLD): a monitor of genome projects world-wide. Nucleic Acids Res.

[B2] Tatusov RL, Fedorova ND, Jackson JD, Jacobs AR, Kiryutin B, Koonin EV, Krylov DM, Mazumder R, Mekhedov SL, Nikolskaya AN, Rao BS, Smirnov S, Sverdlov AV, Vasudevan S, Wolf YI, Yin JJ, Natale DA (2003). The COG database: an updated version includes eukaryotes. BMC Bioinformatics.

[B3] Hsiao W, Wan I, Jones SJ, Brinkman FS (2003). IslandPath: aiding detection of genomic islands in prokaryotes. Bioinformatics.

[B4] Merkl R (2004). SIGI: score-based identification of genomic islands. BMC Bioinformatics.

[B5] Kerkhoven R, Van Enckevort FH, Boekhorst J, Molenaar D, Siezen RJ (2004). Visualization for genomics: the Microbial Genome Viewer. Bioinformatics.

[B6] Sato N, Ehira S (2003). GenoMap, a circular genome data viewer. Bioinformatics.

[B7] Pedersen AG, Jensen LJ, Brunak S, Staerfeldt HH, Ussery DW (2000). A DNA structural atlas for Escherichia coli. J Mol Biol.

[B8] Gibson R, Smith DR (2003). Genome visualization made fast and simple. Bioinformatics.

[B9] Leader DP (2004). BugView: a browser for comparing genomes. Bioinformatics.

[B10] Baerends RJ, Smits WK, De Jong A, Hamoen LW, Kok J, Kuipers OP (2004). Genome2D: a visualization tool for the rapid analysis of bacterial transcriptome data. Genome Biol.

[B11] Rutherford K, Parkhill J, Crook J, Horsnell T, Rice P, Rajandream MA, Barrell B (2000). Artemis: Sequence visualization and annotation. Bioinformatics.

[B12] ACT Home page. http://www.sanger.ac.uk/Software/ACT/.

[B13] ActiveTcl download site. http://tcl.activestate.com.

[B14] PERL Home page. http://www.perl.org.

[B15] Zeeberg BR, Riss J, Kane DW, Bussey KJ, Uchio E, Linehan WM, Barrett JC, Weinstein JN (2004). Mistaken Identifiers: Gene name errors can be introduced inadvertently when using Excel in bioinformatics. BMC Bioinformatics.

[B16] Bray N, Dubchak I, Pachter L (2003). AVID: A global alignment program. Genome Res.

[B17] TIGR sequence data on *Listeria *strains. http://www.tigr.org/tdb/listeria.

[B18] Garcia-Vallve S, Guzman E, Montero MA, Romeu A (2003). HGT-DB: a database of putative horizontally transferred genes in prokaryotic complete genomes. Nucleic Acids Res.

